# Agonism of 4-1BB for immune therapy: a perspective on possibilities and complications

**DOI:** 10.3389/fimmu.2023.1228486

**Published:** 2023-08-17

**Authors:** Shahram Salek-Ardakani, Dirk M. Zajonc, Michael Croft

**Affiliations:** ^1^ Yz Consulting, La Jolla, CA, United States; ^2^ Center for Autoimmunity and Inflammation, La Jolla Institute for Immunology, La Jolla, CA, United States; ^3^ Department of Medicine, University of California (UC) San Diego, La Jolla, CA, United States

**Keywords:** 4-1BB (CD137), agonist, cancer immunotherapy, vaccination, clinical trials, TNFR, autoimmunity

## Abstract

Costimulatory receptors on immune cells represent attractive targets for immunotherapy given that these molecules can increase the frequency of individual protective immune cell populations and their longevity, as well as enhance various effector functions. 4-1BB, a member of the TNF receptor superfamily, also known as CD137 and TNFRSF9, is one such molecule that is inducible on several cell types, including T cells and NK cells. Preclinical studies in animal models have validated the notion that stimulating 4-1BB with agonist reagents or its natural ligand could be useful to augment conventional T cell and NK cell immunity to protect against tumor growth and against viral infection. Additionally, stimulating 4-1BB can enhance regulatory T cell function and might be useful in the right context for suppressing autoimmunity. Two human agonist antibodies to 4-1BB have been produced and tested in clinical trials for cancer, with variable results, leading to the production of a wealth of second-generation antibody constructs, including bi- and multi-specifics, with the hope of optimizing activity and selectivity. Here, we review the progress to date in agonism of 4-1BB, discuss the complications in targeting the immune system appropriately to elicit the desired activity, together with challenges in engineering agonists, and highlight the untapped potential of manipulating this molecule in infectious disease and autoimmunity.

## Introduction

Pioneering work from Byoung Kwon, who discovered 4-1BB (1, 2); Lieping Chen, Robert Mittler, and Ignacio Melero with the first stimulatory antibodies to 4-1BB (3); and the latter together with Tania Watts with over-expression of 4-1BBL (4, 5), established the concept that agonist targeting of 4-1BB can promote responses of T cells and NK cells that are favorable for protecting against tumor growth. Other data initiated by studies from Tania Watts, and from Yang-Xin Fu, Lieping Chen, and Robert Mittler, respectively, further raised the possibility of agonizing 4-1BB to protect against viral infection (6) and to suppress autoimmunity (7, 8). As described in prior reviews (9–14), 4-1BB is an attractive target for immunotherapy firstly because it can be expressed on conventional T cells (both CD8 and CD4) and NK cells, where it’s signals can promote their proliferation and survival, and hence accumulation in numbers, as well as enhance the production of effector molecules such as IFN-γ, TNF, perforin, and granzyme. All of these activities contribute to protective immunity against tumors and viruses, and in the case of certain self-reactive regulatory CTL populations (15–17) might be relevant for augmenting a suppressive immune response that limits autoimmunity.

There are some advantages, but several disadvantages, when considering targeting 4-1BB. 1) 4-1BB is transiently inducible on most of the cells that are desirable to stimulate, including conventional CD8 and CD4 T cells and NK cells, driven primarily by antigen recognition but aided by cytokine action, which is a potential advantage as it might minimize prolonged and off-target activities. 2) Studies of the tumor microenvironment (TME) however promoted the concept that expression of 4-1BB on the aforementioned cells can be negatively regulated, likely from signals from suppressive cytokines or coinhibitory receptors such as PD-1. Thus, together with its naturally brief expression pattern, this presents significant complications in being able to engage 4-1BB on the appropriate cell and to elicit the desired response depending on the context of targeting. 3) Ligation of 4-1BB in isolation on T cells and NK cells may result in some functional effects, such as enhancing their capacity to survive, but its action in driving proliferation or production of effector molecules is primarily as a cosignal, either synergizing on T cells with the T cell receptor when recognizing antigen or synergizing on NK cells with receptors for cytokines such as IL-2, IL-15 or IL-21. Therefore, the full effects of 4-1BB will only be revealed if agonism is provided in these contexts. 4) Other cell types can bear 4-1BB on their membranes, including dendritic cells and macrophages, that may be pro- or anti-inflammatory (18–21), as well as both thymic and peripherally-induced CD4 regulatory T cells (22), and non-hematopoietic cells such as vascular endothelial cells (23–25). Consequently, the activity of several cell types can be elicited by agonist reagents that might or might not be desirable when attempting to augment anti-tumor responses, vaccinate against infectious disease, or treat autoimmunity. 5) The structure of 4-1BB and its mechanism of signaling requires several 4-1BB monomers to be in close proximity and multimerized in order to produce a significant biological effect. This means that to engage it successfully, and strongly stimulate target cells, agonistic biologics need to induce a degree of aggregation on the cell membrane that may not be provided by many simple soluble molecules, such as most conventional antibodies, unless they are clustered on other cells.

Thus, while the concept of stimulating 4-1BB for therapeutic intervention is well grounded, there are considerable hurdles to surmount to be able to do this in a manner that: a) has specificity in targeting the appropriate cell; b) can achieve an appropriate biological effect that is therapeutically relevant; and c) minimizes off-target activity that either results in toxicity, or elicits an immune response that is inappropriate or antagonistic toward the response that needs to be induced. Here, we summarize some of the major clinical efforts agonizing 4-1BB to date in immuno-oncology, provide a perspective on strategies that are being attempted to generate greater specificity in targeting and biological activity, and highlight opportunities in other clinical arenas such as viral vaccines and autoimmunity that have yet to be pursued.

## 4-1BB structure and signaling and agonist biologics

**Figure 1:**
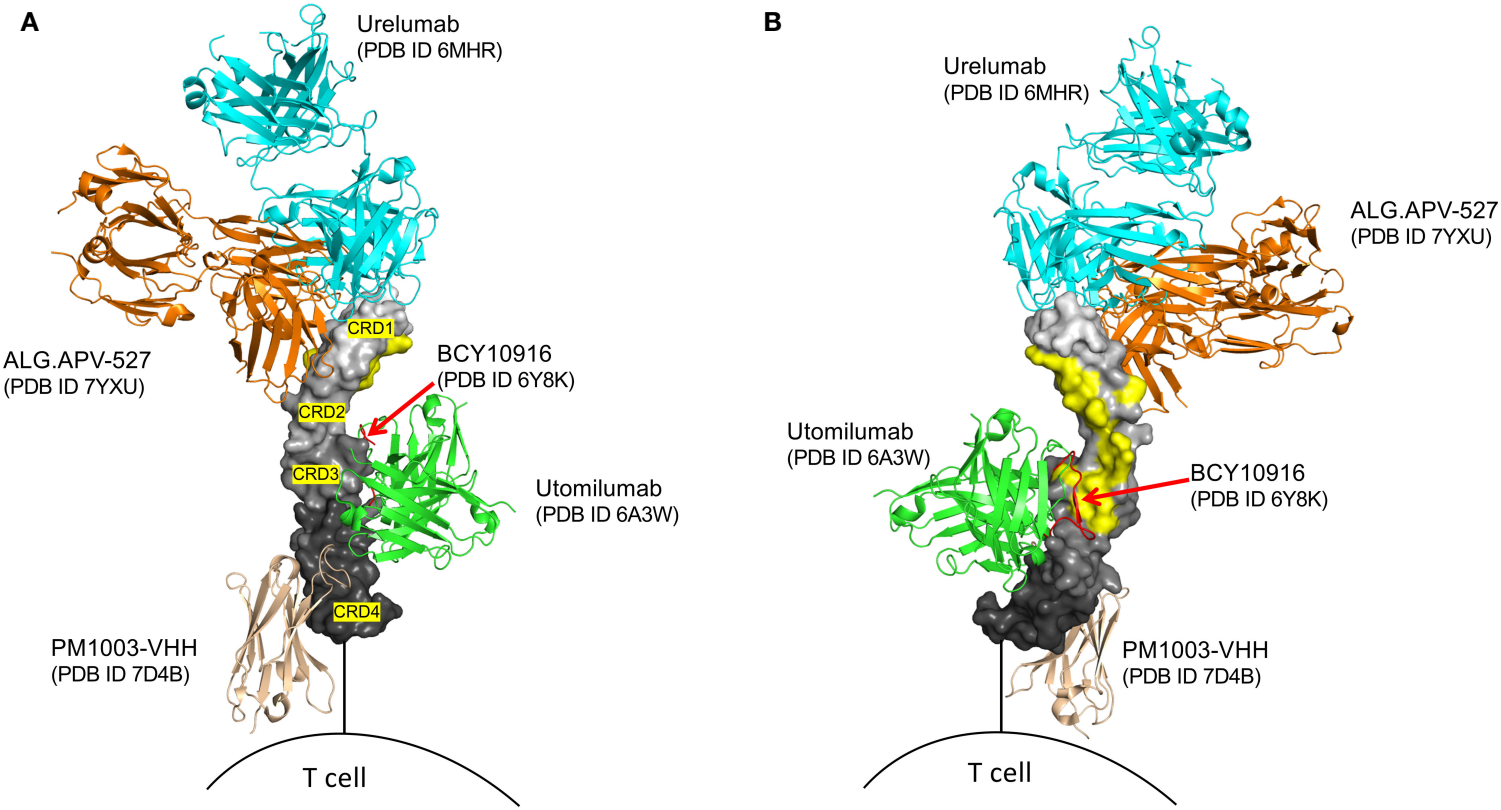
Structural superposition of several agonist modalities targeting human 4-1BB. 4-1BB in graded grey surface representation, with individual cysteine-rich domains (CRD’s) labeled. **(A)** outer view of 4-1BB. **(B)** inner view of 4-1BB, highlighting the 4-1BBL binding sites in yellow. Only utomilumab and the BCY10916 peptide block binding of 4-1BBL to 4-1BB, while urelumab, ALG.APV-527, and PM1003-VHH, allow for simultaneous binding with 4-1BBL. BCY10916 is a close analog of the cyclic 4-1BB binding peptide found in the EphA2/4-1BB bispecific BCY12491.

4-1BB is a monomeric type I transmembrane receptor composed of 4 extracellular cysteine-rich domains (CRD’s), a single-pass transmembrane domain and an intracellular signaling domain (**Figure 1**). Upon binding, *via* the internal face of CRD’s 1, 2, and 3, to its ligand, 4-1BBL (TNFSF9), which is a covalent homodimer in mice (26) and a non-covalent homotrimer in humans (27–29), 4-1BB monomers need to cluster together to allow the intracellular signaling domains to bind effectively to trimeric adaptor proteins, TNF receptor associated factors (TRAF) 1-3. This initiates several downstream signaling pathways, including NF-κB, ERK, and p38 MAPK, which control cellular proliferation, survival, and cytokine production (30). In normal physiology, 4-1BBL is displayed on the surface of cells, and when binding to 4-1BB on another cell, this results in aggregation and allows higher-order clustering of 4-1BB monomers to occur. Since mouse 4-1BBL is only able to dimerize 4-1BB, secondary factors are required to potently cluster monomers, such as by binding Galectin-9 (Gal-9) (31). Gal-9, a tandem-repeat protein, binds to terminal galactose residues of N-linked glycans, and since the N-glycans on 4-1BB are outside the binding site for 4-1BBL in CRD4, Gal-9 is able to secondarily cluster 4-1BB monomers, thereby increasing the valency of the 4-1BB/4-1BBL signaling unit. As the human Gal-9/4-1BB interaction is conserved (31), this might also be important for aiding clustering and signaling on human cells. In addition, human 4-1BB can form covalent dimers, which could also lead to secondary clustering of individual 4-1BBL/4-1BB signaling units (27). Therefore, the 4-1BB signal strength depends on the level of aggregation of 4-1BB monomers, with higher-order multimers of dimers and trimers leading to greater activation of pathways such as NF-κB.

4-1BB agonist targeting can be achieved using the natural ligand, IgG based modalities, or alternate scaffolds such as small cyclic peptides, anticalin’s, and DARPin’s. Its natural human trimeric ligand, when in soluble form, is unlikely to drive the multimerization needed for effective signaling because it cannot be aggregated, and needs to be displayed on an Fc to allow it to exhibit any significant functional agonist activity (32, 33). Similarly, conventional antibodies, because of their bivalent nature, might not engage sufficient monomers for maximizing 4-1BB’s costimulatory signal, even if 4-1BB is naturally clustered on a cell through Gal-9 or covalent interactions. In fact, it is now generally recognized that engagement of the Fc domain of most conventional antibodies to FcγR (particularly FcγRIIB) on a separate cell (e.g. tumor cell or macrophage) is required to cluster enough 4-1BB monomers on a neighboring T or NK cell for effective induction of functional activity (34, 35), an observation shared with agonist antibodies to other TNFRs such as CD40 (36). It is also important to note that the epitope, rather than the binding affinity of 4-1BB antibodies to individual monomers, is another factor that can be important in determining the extent of 4-1BB activation, leading to some exceptions regarding the FcR-dependency, exemplified by urelumab described below. Thus, when considering creating an agonist of 4-1BB, it is not as simple as making a molecule that only binds one 4-1BB monomer.

**Figure 2:**
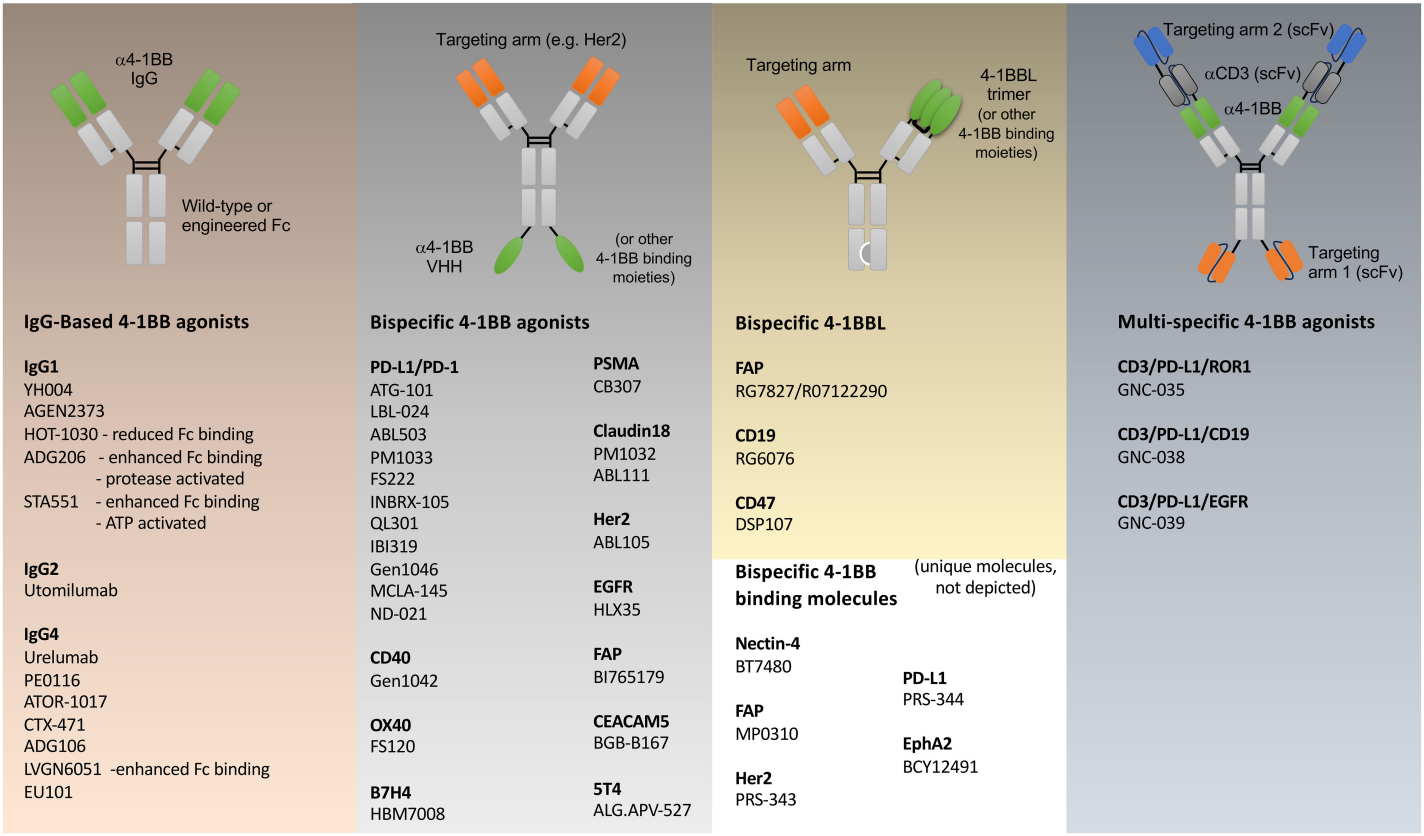
Agonist modalities that have been generated against 4-1BB. Examples of various antibody like constructs targeting 4-1BB are shown, engineered primarily for immuno-oncology, grouped into: simple IgG based biologics; bispecific biologics incorporating binding regions of 4-1BB antibodies in green, that additionally target other functional molecules (PD-1/PD-L1, CD40, OX40, B7H4) or tumor-expressed antigens (PSMA, Claudin18, Her2, EGFR, FAP, CEACAM5, 5T4) in orange; bispecific biologics incorporating 4-1BBL or alternative 4-1BB-binding molecules; and multi-specific T cell engager biologics incorporating binding regions of 4-1BB and CD3 antibodies (grey), and tumor targeting (blue). 4-1BB binding moieties beyond IgG-derived fragments include VHH’s (e.g. HBM7008), 4-1BBL, peptides (BCY12491), DARPins (MP0310) anti-calins (PRS-343,-344). Each model represents an idealized example of the various classes of biologic. Individual reagents that have been produced will vary in structure. Individual structures that are not following an Ig-based scaffold are not depicted and include molecules such as DSP107 (4-1BBL-SIRPα fusion trimers), bicyclic peptides (BT7480), human serum albumin containing fusion proteins (CB307, ND021, NM21-1480). More specific details regarding each agonist modality can be found in Claus et al. (42).

To date all efforts to clinically agonize 4-1BB have been in oncology. Two antibodies were originally produced, urelumab (BMS-663513, IgG4) and utomilumab (PF-05082566, IgG2), that target different domains of 4-1BB (**Figure 1**). Urelumab binds at the tip of CRD1 and does not compete for natural 4-1BBL binding and is a strong agonist. In contrast, utomilumab binds CRD2 and 3 and competes for 4-1BBL binding, thereby reducing the potential of secondary clustering of individual 4-1BBL/4-1BB signaling units, leading to its weak agonist activity (28, 37). Similar observations had been made for anti-CD40 antibodies, where targeting the membrane-distal CRD1 region led to potent agonists, whereas those antibodies targeting CRD2-4, especially those that block CD40L binding, were weak agonists or potent antagonists of CD40 activity (36). Although IgG4 was chosen for urelumab allowing FcγRIIB binding, it is not clear it needs FcR engagement for its activity, however IgG4 and IgG2 (utomilumab) antibodies are also able to engage FcγRIIa and FcγRIIIa, and in addition to 4-1BB clustering, have the possibility to mediate ADCC (38–40) further complicating the development of pure agonist antibodies. While IgG4 can also engage FcγRI, its high affinity to monomeric IgG will result in saturation of the receptor by the high levels of serum IgG, meaning the lower-dosed therapeutic IgG4 antibodies are less likely to engage this FcR (41). As detailed below, in clinical trials of cancer, urelumab, although effective, had issues with off-tumor targeting activities and toxicity, while utomilumab demonstrated weak clinical activity as a monotherapy due to it being a weak agonist. More mono-specific 4-1BB antibodies in addition to urelumab and utomilumab have also been produced with enhanced or reduced FcR binding and other modifications (**Figures 1**, **2**) to allow the ‘optimal’ level of 4-1BB engagement for agonism, but it is still not clear what characteristics an antibody needs to possess to provide this optimum. Furthermore, none of these approaches address the issue of specificity or selective agonism, i.e. being able to target the right cell type in the right location. Additional approaches have then been deemed desirable, leading to the development of a wealth of second-generation modalities aimed at maximizing agonism while engendering specificity. These are described in detail in several excellent recent reviews of cancer immunotherapy (42, 43) and are listed in **Figure 2**, and further discussed in general terms below.

One approach is tumor-targeting, covering both heme malignancies (CD19) and solid tumors (PD-L1, Her2, FAP, EGFR, PSMA, Cldn18.2, Nectin 4, B7H4, CEACAM5, 5T4, EphA2). Most tumor-targeted modalities are bispecific antibodies where a single arm engages the tumor-associated antigen (TAA) on the tumor cell, while the other arm targets 4-1BB, with the aim of only activating tumor-infiltrating lymphocytes (TILs) present in the tumor microenvironment (TME) (44–50). Another concept is to target 4-1BB exclusively on T cells through the simultaneous binding of two T cell proteins, such as 4-1BB and OX40 (51), or 4-1BB and PD-1 (52). Both PD-1 and OX40 are upregulated on antigen-responding T cells, allowing more specific engagement of 4-1BB on only subsets of T cells that hopefully are relevant for tumor elimination. A third approach combines 4-1BB costimulation with T cell engagers (TCEs). Traditionally, T cell engagers bridge T cells and tumor cells *via* simultaneous TAA and CD3 binding, leading to the activation of all T cells regardless of their antigen specificity (53). This modality circumvents the need for TCR recognition of an MHC-presented peptide and has been successfully used in heme malignancies. However, solid tumors pose a challenge for T cell engagers, either due to the lack of T cell infiltration, the lack of a durable and potent T cell response, or T cell exhaustion due to the immune suppressive microenvironment that limits T cell cytotoxicity. In an attempt to overcome some of these challenges, 4-1BB antibodies have been combined with a TCE in a single molecule (42, 54). Several multi-specific TCE’s that contain 4-1BB antigen-binding have been produced (**Figure 2**), with GNC-035 being in clinical trials for breast cancer (NCT05160545) and hematologic malignancies (NCT05104775) and GNC-038 in trial for central nervous system lymphoma (NCT05485753). Although pre-clinical data has not been published, the design of these molecules are complex, containing 2 binding domains each against CD3, 4-1BB, PD-L1, and ROR1 or CD19. In summary, clearly novel, and highly complex, protein engineering ideas are fueling the field of 4-1BB agonism, but which is the best tactic is still to be determined, and one that might vary depending on the cellular target and goal in terms of disease modification.

## Clinical targeting of 4-1BB in cancer

The rationale for agonizing 4-1BB in cancer is strong, given the ability of 4-1BB to drive CD8 T cell and NK cytotoxic activity (3–5, 10, 12), and was spearheaded by the first-generation agonist antibodies, urelumab (BMS-663513) and utomilumab (PF-05082566) that were evaluated both as monotherapies and combined with other therapies (28, 37, 55–62). Urelumab monotherapy demonstrated activity, but modest clinical response rates have prompted the exploration of combination therapies. In a trial of several B cell lymphomas, urelumab monotherapy achieved objective response rates (ORR) of 6-17% and disease control rates (DCR) of 19-42% depending on the tumor type, and the combination of urelumab with rituximab had improved outcomes of 10-35% ORR and 24-71% DCR (63). Utomilumab treatment alone has shown less apparent activity in clinical trials as a monotherapy, although it is difficult to compare the two antibodies as the types of cancer targeted and patient populations treated have varied. For example, a phase 1 trial in patients with advanced solid tumors reported an ORR of 4% in 53 patients, but stable disease in 25% and a disease control rate (DCR) of 28% (60), and a phase 1 trial in advanced melanoma or NSCLC reported ORRs of 2% and 0%, respectively, although again 23-50% of patients showed stable disease (62). Utomilumab in combination with pembrolizumab in 23 patients with advanced solid tumors gave an ORR of 26% and a DCR of 70% (64), and when combined with rituximab, an ORR of 21% in patients with non-Hodgkin lymphomas was reported (61). However, although the combination treatments displayed more efficaciousness than the anti-4-1BB antibodies alone, it was not clear if response rates were significantly different than historically seen with the partner drugs.

Urelumab was associated with a higher incidence of immune-related adverse events (irAEs) than utomilumab, including cytokine release syndrome (CRS), immune-mediated colitis, hepatotoxicity, and dermatologic reactions (58). Deaths associated with urelumab treatment have also been reported, primarily due to severe cytokine release syndrome and hepatotoxicity. Although the exact mechanisms underlying the observed toxicities are not fully elucidated, preclinical data with agonists with similar properties to urelumab have shown that T cells are required, and associated with anti-4-1BB upregulating IFN-γ, TNF, and IL-6, and systemic inflammation and organ damage. This is presumably as a result of presentation of autoantigens that might be available in various tissues, given that 4-1BB ligation on T cells in the absence of an antigen-induced T cell receptor signal is unlikely to result in significant cytokine production. Also, studies of the liver have suggested that 4-1BB can be expressed on infiltrating monocytes and tissue-resident Kupffer cells, which when ligated can further contribute to inflammatory cytokine production, enhance antigen presentation to T cells, and lead to hepatotoxicity. Furthermore, engagement of anti-4-1BB by FcγR expressed on these myeloid cells or other similar cells has been suggested to be critical for toxicity, which may additionally amplify the T cell effects, as well as lead to other direct or indirect activities such as promoting the expression of Fas on liver cells, rendering them susceptible to Fas-mediated apoptosis (21, 40, 65–68). Considering the challenges associated with toxicity, the development of urelumab as a monotherapy has largely been discontinued. Similarly, the development of utomilumab as monotherapy has also been discontinued, driven by strategic decisions and the pursuit of more efficaciousness.

One idea put forward is that alternative dosing regimens for antibodies to 4-1BB may achieve a better balance between therapeutic efficacy and manageable toxicities (40, 42, 43, 69). These regimens include dose escalation, dose fractionation, or intermittent dosing. However, while dose optimization is conceptually viable, practical and commercial feasibility presents significant challenges. The inherent heterogeneity among patients and cancer types and the complex interplay of various factors influencing treatment response make establishing universally appropriate dosing regimens challenging. Tailoring dosing regimens individually may require substantial resources, including comprehensive patient profiling, ongoing monitoring, and dose adjustments, and be time-consuming, costly, and impractical. Additionally, changing the dose does not alter the intrinsic agonist activity of an individual antibody on a cell, based on its 4-1BB epitope binding and affinity, and does not circumvent the potential for off-target effects due to the expression of 4-1BB on cells in other organs or on suppressive cells such as Treg. Thus, while dose optimization may have some value, it is unlikely to strongly lead to greater efficacy and improved safety while accounting for the biological, logistical, and economic considerations.

Nevertheless, the knowledge gained from these early studies has provided valuable insights for developing next-generation 4-1BB biologics that specifically aim to overcome limitations in agonism and decouple efficacy from toxicity (**Figure 2**). Moreover, because 4-1BB can be expressed on cells, such as endothelial cells, dendritic cells, macrophages, and regulatory T cells, that might counter anti-tumor activity, attempts are being made to provide greater specificity in cell targeting (40, 70–78). While the merits of these ideas are discussed below in general terms, most of the second-generation agonists mentioned in **Figure 2** are still in early clinical development with only a small amount of data from phase I studies being reported at present in peer-reviewed publications (49, 50, 79, 80).

### Modified binding affinity and specificity

One approach being explored involves the development of 4-1BB agonists with modified binding characteristics (affinity or epitope specificity) (43, 74, 81). Preclinical studies have provided some insights into the potential success of this approach. By carefully screening antibodies with broad epitope coverage and fine-tuning their binding affinity through site-directed mutagenesis, enhanced antitumor immune responses and reduced immune-related adverse events have been reported in some animal models (74, 81). However, a major challenge, both theoretically and practically, is the idea of identifying the optimal or “magic” epitope on 4-1BB that might selectively activate the desired signaling pathways in the desired target cell without either triggering excessive immune activation or associated toxicities. The intricate nature of 4-1BB receptor regulation and ligand interaction makes this task complex and potentially futile. It has been suggested that retaining 4-1BBL binding by not interfering with its binding sites, as with urelumab and ALG.APV-527 (**Figure 1**), will aid agonism, which is logical given the need for 4-1BB monomers to cluster and potentially for the clustering of 4-1BBL-organized trimers. However, it is unlikely that this will circumvent off-target activities. Moreover, varying the binding epitope of 4-1BB antibodies to be outside of the 4-1BBL-binding region (e.g. in CRD1 or 4) and with the goal of inducing different functional outcomes, is akin to threading a needle, especially given that 4-1BB expression on any individual cell can vary, both in density and in intrinsic clustering from covalent interactions or *via* coreceptor proteins such as Galectin-9. Furthermore, preclinical studies of these reagents in mice are complicated given differences in 4-1BB receptor expression and clustering between mice and humans, and that mouse 4-1BBL is a dimer and not a trimer which will influence overall signaling and functional outcomes.

### Fc engineering

Another approach to generate a better agonist has involved removing the Fc domain (81, 82) or altering the antibody isotype to one with reduced binding to FcR (83), to try to circumvent toxic side effects such as liver damage thought dependent at least in part on clustering of anti-4-1BB on FcR on Kupffer cells (35). However, it is important to note that these modifications may impact the antibody’s effector function and antitumor activity. A different approach is to modify the Fc glycan structure of the 4-1BB antibody, e.g. through afucosylation, which reduces binding to FcγRIIIa, and has been reported to decrease liver toxicity while maintaining antitumor activity in mice (40, 84). Point mutations in the Fc region also can modulate the antibody’s affinity for Fc receptors, such as decreasing binding to FcγRIIB, which was reported to reduce the risk of thrombocytopenia while preserving antitumor activity (85). Half-life extension without the use of the Fc region is an additional strategy where the antigen binding region of the 4-1BB antibody is linked with another protein, such as serum albumin, altering the antibody’s pharmacokinetics, including its half-life. Again, in mice, this has been reported to result in enhanced antitumor activity while minimizing toxicity (78). However, it is important to acknowledge the complexities and differences that may affect the translatability of these findings to humans (35). Humans exhibit distinct patterns of Fc receptor expression on immune cells compared to mice, and humans possess FcRs, such as FcRn, with different affinities for various IgGs compared to mice. Fc glycosylation also differs between mice and humans, which can impact the degree of activation induced by Fc-engineered antibodies. Given these variabilities, achieving a transformative impact with Fc-engineered 4-1BB antibodies in the clinic remains challenging, and evaluating the translatability of mouse findings likely will require humanized mouse models and conducting thorough pharmacokinetic and pharmacodynamic studies in human subjects. Studies in non-human primates, while essential, also have their drawbacks. While FcγRs are similar to those in humans, their distribution differs (86). In addition, other differences also confound interpretation of antibody effects, as illustrated by urelumab exhibiting minimal toxicity in non-human primates as opposed to humans, possibly related to the affinity of urelumab for macaque 4-1BB being lower than for human 4-1BB.

### Selective agonism

Other arenas are aiming to create strategies for selective agonism of anti-4-1BB drugs, to specifically activate 4-1BB signaling pathways only in the TME and on desirable target cells, such as CTL, while minimizing off-target effects and toxicity. These may hold the greatest chances for success. As mentioned above, bi-specific antibodies designed to bind 4-1BB and a tumor antigen simultaneously, such as Her2, EGFR, or CEACAM5, should result in the selective activation of 4-1BB pathways in the TME (43, 81). Based on preclinical and early clinical studies, CD19 and CD20 bi-specific antibodies have potential for selectively enhancing 4-1BB signaling in B-cell malignancies (44). Another approach involves using antibodies engineered to be activated exclusively in the tumor microenvironment. These antibodies are designed with a tumor-specific trigger, such as enzymatic activation or pH-dependent conformational changes, initiating their activation and 4-1BB engagement in the TME (77, 87, 88). Furthermore, targeted delivery systems are being developed, using nanoparticles, liposomes, or other conjugates specifically designed to reach the tumor site (89). Despite this, their efficacy may be limited by the restricted distribution and penetration of the treatment within the tumor. Tumors often exhibit heterogeneous characteristics, including variations in antigen expression, immune cell infiltration, and vasculature. Consequently, exclusively targeting 4-1BB agonists to the tumor may only partially engage all relevant immune cells or tumor subpopulations.

Another limitation of these approaches is the likelihood that 4-1BB agonists might also need to have important activity in lymphoid tissues, particularly tumor-draining lymph nodes. 4-1BB signaling in lymph nodes can reactivate non-exhausted memory T cells, or even new naive T cells, specific for tumor neoantigens. Therefore, restricting 4-1BB agonist activity solely to the TME could potentially limit the full activation and expansion of tumor protective T cells. As 4-1BB is primarily induced on T cells by antigen recognition, in this case there is an argument for a vaccine-type approach, using neoantigen administration in combination with 4-1BB agonism, to effectively engage relevant T cells outside of the TME. Additionally, the restricted expression of 4-1BB in the tumor microenvironment is an important consideration. It is clear that 4-1BB expression is often confined to a small subset of tumor-resident T cells (most often less than 20%), and many of these can be Treg, or it is only seen in certain tumor types and not others, while most conventional CD8 and CD4 T cells within tumors may lack 4-1BB (90–94). To overcome this limitation, combination strategies are likely needed to try to induce 4-1BB expression on a broader population of T cells within the tumor. For example, immune checkpoint inhibitors that block coinhibitory receptors, such as PD-1 or CTLA-4, have resulted in enhanced 4-1BB expression on T cells (95–99). Combining checkpoint inhibitors with tumor-targeted agents in bi-specific or multi-specific formats could increase the number of T cells capable of responding to 4-1BB agonism (45, 49, 52, 75, 100). Other immunomodulatory agents, including cytokines like IL-12 and IL-15, or immune stimulatory molecules such as TLR agonists (101–106), also can modulate directly or indirectly 4-1BB expression on T cells, and are potentially good approaches for combination therapy. However, the optimal strategy and their efficacy for increasing the availability of 4-1BB on the appropriate cell type, not the inappropriate one, require further investigation and validation through preclinical and clinical studies.

### CD3 T cell engagers

A variant example of selective agonism is the creation of CD3 T cell engagers (TCEs) that incorporate antibody binding regions of 4-1BB with CD3, and checkpoint blockade (PD-L1), and a tumor target such as CD19 or EGFR (**Figure 2**), with the aim of only engaging 4-1BB on a T cell in the TME (53, 107). While TCEs are an interesting concept, published work on any incorporating 4-1BB binding is limited, and such a multivalent modality has many potential drawbacks and still risks having off-tumor and off-target effects. A major consideration is whether the targeting arms specific for a TAA (depending on how they are displayed in the construct) are sufficient for ensuring localization to the TME. As such, this modality might have a similar liver toxicity risk as traditional 4-1BB antibodies. Similarly, would both 4-1BB targeting arms be sufficient for strongly activating 4-1BB expressing cells. If they are not, this may negate toxicity but also mean little efficacy. Moreover, will T cells be activated without binding to the TAA, since the TCE could simultaneously engage two CD3 molecules and two 4-1BB molecules on the same T cell. In addition, the molecule would have to be engineered to favor cis-binding of both 4-1BB and CD3 on the same T cell, rather than providing signal 1 to one T cell and signal 2 to the other T cell which would ultimately reduce the potency of the molecule (108). Thus, while the notion of complex TCEs providing specificity, targeting, and limiting toxicity, all in one, is good, generating the appropriate construct that exhibits all of the relevant activities may be very challenging.

### Other combination therapies

Yet another approach to maximize the potential of 4-1BB agonism is to combine 4-1BB antibodies with other therapies. Preclinical or clinical studies have shown promise with various combinations, including chemotherapy or radiation treatment, T cell engagers, CAR T cells, cytokines such as IL-2, antibodies to checkpoints such as anti-PD-1, or antibodies to other costimulatory molecules such as anti-OX40 (43). It is important to note that the incidence and severity of adverse events have either been found to, or are likely to, vary depending on the specific combination, dosing, patient population, and prior treatment history, but most importantly that adverse events will be similar to those with 4-1BB antibody monotherapy, with none of these combinations at present mitigating the off-target effects of anti-4-1BB or providing selective agonism. Perhaps future evaluation of the second-generation bi-specific and multi-specific reagents with some of these combinations will provide the level of agonism desired to harness the potential of 4-1BB antibodies to enhance antitumor immune responses while minimizing toxicities.

### Additional considerations to maximize 4-1BB agonism in cancer

As alluded to before, one major restriction is whether 4-1BB is expressed on the cell type(s) most desirable to target for cancer immunotherapy. Biomarker-based patient selection approaches can help to identify specific biological markers associated with improved response rates (109). Obviously, the most promising biomarker is the expression of 4-1BB itself in the tumor microenvironment and tumor draining lymph nodes. Studies in patients with certain tumors have demonstrated that high levels of 4-1BB expression are associated with a higher response rate to therapy (109–111). Anecdotally, it has also been suggested that the presence of TILs will be associated with an improved response to 4-1BB-targeted therapies, and patients with a higher density of TILs may exhibit a higher response rate to 4-1BB agonism. In addition, biomarkers such as PD-L1 or IFN-γ appear to indicate the presence of an active antitumor immune response that could be further enhanced by 4-1BB-targeted therapy, and patients with high levels of tumor IFN-γ may then demonstrate a higher response rate. Although incorporating biomarkers that predict treatment response is crucial, it is equally important to identify biomarkers that might predict the risk of toxicity. However, identifying and validating reliable biomarkers requires extensive research and clinical studies and the complexity of the immune system and TME presents a challenge in accurately assessing treatment response and predicting toxicities. Moreover, the heterogeneity of patient populations and tumor types poses a challenge for biomarker-based patient selection, as the predictive biomarkers may vary among different cancer types. Technological advances, such as high-throughput sequencing and proteomics, artificial intelligence, and machine learning algorithms, offer opportunities to identify novel biomarkers as well as provide longitudinal insights into treatment response dynamics. By leveraging technological advancements, collaborative efforts, and innovative approaches, the future development of biomarker-based patient selection holds tremendous potential to optimize 4-1BB-targeted therapies and improve patient response.

## Agonizing 4-1BB in viral vaccines

As well as cancer immunotherapy, an obvious but still underappreciated application of agonizing 4-1BB is in vaccination against viral infections, given the importance of T cells and NK cells in protective immunity. However, many of the same issues discussed above also apply in this arena. Targeting 4-1BB therapeutically during active acute infections has many practical challenges, not least of which are treating patients within the critical short time period when 4-1BB will be induced on virus-responding T cells or virally activated NK cells and the feasibility of administering an agonist such as an antibody to those patients. Also, the potential for driving unwanted virus-induced pathology by excessively triggering CTL activity during an active infection, as is naturally seen in some patients with SARS-CoV-2 or influenza virus, is a true risk that would limit the therapeutic use of 4-1BB agonists.

However, incorporating an agonist into a prophylactic vaccine has much appeal. Following the initial demonstration that an agonist antibody to 4-1BB could increase the frequency of LCMV-reactive CD8 T cells in mice vaccinated with an LCMV peptide (6), a number of other studies with vaccine protocols using viral peptides, live or attenuated viruses, or DNA plasmid vectors encoding viral proteins (**Table 1**), have demonstrated a similar phenomenon with agonist antibodies to 4-1BB (112–119). This has been seen with responses to influenza virus, HCV, HSV, Friend virus, VACV, RSV, and CHIKV in mice, not only promoting a greater magnitude of acute CD8 and/or CD4 effector T cell responses but also enhancing protective T cell memory against re-infection, as well as in some cases broadening the repertoire of anti-viral specific T cells. Other studies (**Table 1**) have used 4-1BBL to deliver the agonist signal in mice or tested *in vitro* with human cells, with 4-1BBL either incorporated into adenoviral or other DNA vectors for direct injection, or with plasmid transfection into monocytes, fibroblasts, or dendritic cells for cell therapy, all with similar results on enhancing T cell immunity to viral antigens (120–128). In total, these results have then created a very strong argument that agonizing 4-1BB would be highly useful and effective against infection with multiple viruses if integrated into a vaccination strategy. Moreover, the ability of 4-1BB to drive persistently high numbers of memory T cells, including those resident memory cells that accumulate in peripheral tissues (101, 128, 129), and to overcome defects in T cell immunity associated with aging (118, 130), is highly relevant given the current conversations around persistence of T cell memory and effectiveness of COVID mRNA vaccines in adults and older people.

**Table 1:** Summary of major studies demonstrating that agonist antibodies to 4-1BB, or forced expression of 4-1BBL, can enhance T cell priming and memory T cell responses, and protective immunity, in vaccine protocols with virus infection, or immunization with viral peptides or vectors encoding viral antigens.

Virus or viral antigen	Organism or cells	4-1BB agonist and delivery	Functional Effect of Stimulating 4-1BB	Reference
LCMV NP peptide	Mice	Antibody i.p	Increased primary splenic NP-specific IFNγ CD8 T cells	Tan, 2000
Influenza PR8	Mice	Antibody i.p	Increased # primary lung flu-specific CD8 T cells and cytotoxicity	Halstead, 2002
Influenza HKx31	Mice	Antibody i.p	Increased # primary and memory splenic flu-specific CD8 T cells and cytotoxicity	Bertram, 2004
Influenza M1 peptide; EBV BMLF1 peptide	Human	Adenovirus encoded 4-1BBL in monocytes	Increased flu-specific IFNγ, TNF, and cytotoxic memory CD8 T cells	Bukczynski, 2004
HIV env, nef, gag peptides	Human	Adenovirus encoded 4-1BBL in monocytes	Increased # memory HIV-specific CD8 T cells, and cytotoxicity	Bukczynski, 2005
Adenovirus encoded HCV-NS3	Mice	Antibody i.p	Increased NS3-specific CD4 IFNγ, and CD8 cytotoxicity, and protection against HCV infection	Arribillaga, 2005
HSV-1	Mice	Antibody i.p	Increased # primary and memory LN HSV gB-specific CD8 T cells and cytotoxicity, and protection against HSV-1 reinfection	Kim, 2005
HIV A/E gag/pol	Mice	Fowlpox virus encoded 4-1BBL i.m	Increased # splenic HIV-specific CD8 T cells and IFNγ	Harrison, 2006
HCMV pp65	Human	pcDNA3 encoded 4-1BBL in fibroblasts	Increased # HCMV-specific CD8 T cells	Waller, 2007
FV	Mice	Antibody i.p	Increased # primary splenic FV-specific CD8 T cells, IFNγ and cytotoxicity, and reduced virus replication	Robertson, 2008
pGA1 and MVA encoded HIV gag/pol/env	Mice	Antibody i.p and pGA1 encoded 4-1BBL i.m	Increased # primary and memory HIV-specific CD4 T cells and IFNγ CD8 T cells	Ganguly, 2010
Adenovirus encoded influenza NP	Mice	Adenovirus encoded 4-1BBL i.m and i.n	Increased # primary and memory lung, splenic, and LN influenza-specific CD8 T cells and cytotoxicity, and protection against influenza infection	Moraes, 2011
VACV-WR; VACV-Lister; VACV B8R, N2L and B16R peptides	Mice	Antibody i.p	Increased # primary and memory splenic and lung VACV-specific CD8 T cells and TNF and IFNγ, and protection against VACV infection	Zhao, 2012
pcDNA3 encoded HIV gag	Mice	pcDNA3 encoded 4-1BBL and SF protein D i.m	Increased # primary and memory HIV-specific IFNγ CD8 T cells	Kanagavelu, 2012
RSV M2 peptide, anti-CD40, polyIC	Mice	Antibody i.p	Increased % blood and lung RSV-specific CD8 T cells, IFNγ, and cytotoxicity, and increased protection against lung RSV infection	Lee, 2014
Adenovirus encoded HIV gag	Mice	Adenovirus encoded 4-1BBL in dendritic cells i.v	Increased # HIV-specific CD8 T cells	Wang, 2015
Adenovirus encoded influenza NP	Mice	Adenovirus encoded 4-1BBL i.n	Increased # stable lung memory influenza-specific CD8 T cells, IFNγ and cytotoxicity, and protection against influenza infection	Zhou, 2017
CHIKV; MAYV	Mice	Antibody i.p	Reduced T cell dependent primary splenic and LN GC B cells and viral RNA	Hong, 2019

As yet, no clinical trials of viral vaccination have attempted to agonize 4-1BB. One study more than 10 years ago tested an agonist antibody to 4-1BB in NHP given an intramuscular SIV DNA vaccine (131). This resulted in an increase in the SIV-specific CD8 T cell response and a decrease in viral titers after the SIV challenge, as predicted. In contrast, another study with 4-1BBL in DNA plasmid or viral vectors again showed increased CD8 T cell responses in mice, but in a limited study in NHP, mixing a plasmodium antigen-encoding vector with another vector encoding 4-1BBL, intramuscularly, resulted in no enhancement of IFN-γ producing cells (132). The latter could have reflected a need for 4-1BBL to be co-expressed in the same vector with antigen or the lack of another adjuvant activity. Although little work has since moved away from the mouse, this field is particularly ready and appropriate for translation to humans if the right vehicle and adjuvant system can be found to agonize 4-1BB, especially given the recent focus and success of mRNA vaccination.

Off-target effects are again a potential and likely problem with agonist antibodies, as illustrated by repeated injections of anti-4-1BB into HBV-transgenic mice resulting in hepatitis, fibrosis, and liver cirrhosis, mimicking liver disease during natural chronic HBV infection (67). This is similar to the issues with cancer immunotherapy. However, in the case of viral vaccines, the development of bi- or multi-specific antibodies to surmount targeting the wrong cell type in the wrong location is far more of a challenge than when making use of the TME and tumor-associated proteins, given that viruses can replicate in multiple organs and many cell types. Thus, promoting the expression of 4-1BBL encoded in DNA or mRNA with viral antigen would be preferable to minimize off-target adverse events, given that it is likely that subcutaneous, intradermal, or intramuscular administration of these vaccines would focus 4-1BBL more specifically to the cells that present viral peptides directly to T cells. Some studies in mice comparing anti-4-1BB to 4-1BBL delivered in a DNA vaccine support the idea that vaccines encoding 4-1BBL could limit such adverse events (124). 4-1BBL-transduced dendritic cells or macrophages bearing viral antigens used in adoptive cell vaccines, or extracellular vesicles (EVs) derived from these cells, could be an alternative to DNA or mRNA delivery. This may again create more specificity in delivering 4-1BB signals to the appropriate cells, but these methods are not yet attractive for large-scale vaccination efforts.

What would be the best vaccine centered around 4-1BBL is still to be determined. Agonists to 4-1BB were initially shown to perform much better in preclinical studies when combined with the TLR3 adjuvant poly I:C in terms of their ability to increase the magnitude of the T cell response that can persist (101). However, whether this TLR is the preferred partner to maximize 4-1BB activity is not clear given that other studies have reported apparent synergies with ligands of TLR4, TLR7/8 or TLR9 (103, 104, 106). IL-15 can also induce or prolong 4-1BB expression on T cells, suggesting its incorporation into a vaccine would help with enhancing or prolonging 4-1BB signaling (102, 105), and IL-7 can promote TRAF1 levels in T cells which would further aid the ability of 4-1BB to signal (133). As detailed above, synergistic activities of agonizing 4-1BB and blocking PD-L1 have also been reported in several tumor studies, leading to the current bispecific constructs reviewed earlier, and this synergy has additionally been seen in models of chronic virus infection (133, 134). Therefore, DNA or mRNA viral antigen vaccines encoding 4-1BBL with one or several of these factors are likely to be more effective than simply combining 4-1BBL with the viral antigen alone. One novel way of delivering 4-1BBL was reported, constructing what was termed a synTac, a dimeric Fc fusion protein incorporating 4-1BBL with MHC complexes of peptides of HIV or CMV (135). This could represent a further approach to increase specific targeting of relevant T cell populations whilst minimizing off-target effects, and such constructs can be further modified by adding cytokines such as IL-7 and IL-15, or TLR ligands, to complement the adjuvant activity.

## Agonizing 4-1BB in autoimmunity

Lastly, an unexpected finding that has been revealed in the field of 4-1BB agonism is the ability to shut off or limit autoimmune and other inflammatory reactions. This has been seen with agonist antibodies injected into murine models of SLE (7, 8, 136), MS (31, 137), RA (16, 138), conjunctivitis (139, 140), IBD (141), uveitis (142, 143), asthma (31, 144, 145), type I diabetes (146), chronic GVHD (147), diet-induced obesity (148), psoriasis (149), and Sjogren’s syndrome (150). In general, suppression driven by anti-4-1BB has been seen during the initiation phases of the disease rather than with therapeutic intervention during active disease, although in some models, therapeutic activity has been noted (8, 138).

Two primary mechanisms of 4-1BB-driven suppression have been suggested, either promoting the accumulation and activity of conventional CD4^+^Foxp3^+^ Treg that can express 4-1BB, or more in-line with the tumor and virus literature on 4-1BB, inducing the differentiation or reactivation of CD8 T cells, either into CTL or a type of CD8^+^ Treg that makes IFN-γ and can suppress the normal inflammatory response of CD4 T cells or B cells (16, 22, 140, 141, 143, 144, 151–154). Other suppressive activities such as directly driving death of pathogenic effector T cells, expanding MDSC, or promoting regulatory activities in dendritic cells, have also been suggested (19, 20, 142, 147, 155).

While the literature on this aspect of 4-1BB agonism is extensive, translating the research to humans is an opportunity that has not been pursued as yet, primarily because there are many targeting hurdles to overcome. A major concern if contemplating using agonist antibodies in patients with active autoimmune or inflammatory disease is whether stimulating 4-1BB could trigger pathogenic effector CD4 or CD8 T cells or other inflammatory cell types such as pro-inflammatory macrophages and exacerbate the specific disease. This has been seen in some mouse disease models (156–158), and would be a problem with an antibody to 4-1BB as well as with simple injection of 4-1BBL in soluble or vector form. Engineered bi- or multi-specific antibody constructs are again unlikely to circumvent this issue unless a targeting partner can be found that is only expressed on the regulatory/suppressive cell type whose activity needs to be enhanced. As discussed above, this could be a CD4^+^Foxp3^+^ Treg, a regulatory DC or MDSC, or a CD8^+^ Treg that can kill or suppress pathogenic effector cells. At present, it is not clear if such markers exist or can be found that truly distinguish these cells from non-regulatory cells. However, continued screening efforts with single-cell RNA-seq and CITE-seq might be able to reveal a protein or proteins whose targeting could be incorporated into a second-generation agonist.

Another potential path forward that, on paper, is more feasible is a vaccine-like strategy that incorporates an antigen into an mRNA or DNA vaccine utilizing 4-1BBL. This could again minimize off-target effects and focus 4-1BBL on cells that present antigen directly to regulatory CD4 or CD8 T cells. The question here is whether a relevant antigen or peptide epitope can be defined that would only be recognized by the Treg. A significant literature exists on what sometimes have been termed Tregitopes. These can be epitopes of proteins that have been argued to be specifically recognized by thymic Treg or regulatory CD8 T cells, and have been described to range from peptides in the Vβ regions of TCRs of autoreactive T cells to peptides presented on non-classical MHC molecules, to conserved repeat regions in the Fc domain of IgG (15, 17, 159–163). If these can be shown in humans to truly be specific for pre-existing Treg or for driving the differentiation of newly formed Treg, this can potentially harness the utility of agonizing 4-1BB in an mRNA or DNA formulation used in prophylactic or therapeutic vaccination.

An easy alternative to *in vivo* agonism of 4-1BB, of course, could be to exploit the ability of 4-1BB signals to expand Treg or CTL *in vitro* (164–168). These could be used in adoptive cell therapy of autoimmune disease, although this is not as attractive for widespread therapeutic treatment. In this case, knowledge of a relevant autoantigen for CD4 Treg, and a relevant antigen for regulatory CD8 T cells, such as a peptide of a dominant Vβ TCR expressed on autoreactive CD4 T cells or a peptide presented on non-classical MHC recognized by suppressor CD8 T cells, would again likely be needed to allow specific targeting and inhibition of the T cells that drive autoimmunity or other inflammatory diseases (169). Engineering Treg with specific TCRs of autoantigens, if they can be identified, would be another option. Lastly, attempts are already underway to use CAR Treg therapy for autoimmune disease, along with identifying specific antigens that can be used to mobilize these Treg (169), which could be further expanded in number with agonists to 4-1BB. Although only indirectly relevant for the current discussion, incorporation of the intracellular domain of 4-1BB has already been established to be beneficial in such cells.

## Concluding remarks

The fact that interest in 4-1BB as a therapeutic target has persisted and even expanded in the past few years, given the less-than-compelling results in clinical trials with agonist antibodies, is a testament to the potential that has been raised for this molecule from basic research in preclinical studies. Although we have provided opinions for and against strategies that might or might not be fruitful in oncology, and also in infectious disease and autoimmunity, our enthusiasm for 4-1BB agonism is still extremely high. The use of 4-1BB agonist antibodies in cancer treatment has shown promise in enhancing antitumor immune responses, but several challenges and limitations must be addressed. Optimal dosing and treatment regimens are a primary challenge. Balancing immune activation and toxicity avoidance is complex. Monitoring and managing treatment-related toxicities are crucial. Biomarker-based patient selection approaches, including predictive biomarkers of treatment response and toxicity, are important to understand the heterogeneity of patient populations and how individual groups will benefit. The complexity of the tumor microenvironment and its immunosuppressive mechanisms pose challenges, as does the complexity of autoimmune and inflammatory disease. Advances in technology and our understanding of immune system dynamics can optimize treatment strategies, and integrating immune cell phenotyping and genetic profiling can aid in patient selection and personalized treatment approaches.

Ongoing research aims to identify novel 4-1BB agonists with next-generation bi-specific or multi-specific platforms targeting the 4-1BB pathway together with other pathways, and applying predictive modeling and machine learning algorithms can assist in tailoring therapy to individual patients. Thus, despite the many challenges, future development in 4-1BB agonist biologics holds substantial potential. The varied concepts proposed in multi-specific targeting through protein engineering, in academia and especially in industry, demonstrate the wealth of talent available to bypass and solve the complexities of the immune system. With good science, trial and error with the many great ideas that have arisen in this area, and some fortune, we remain confident that targeting 4-1BB will ultimately be productive and therapeutically efficacious.

## Author contributions

All authors equally contributed to writing and editing the manuscript. All authors contributed to the article and approved the submitted version.
